# Severe hypocalcemia in a patient with extensive cranial Paget disease: a diagnostic challenge

**DOI:** 10.1210/jcemcr/luag100

**Published:** 2026-05-27

**Authors:** Yagmur Goksoy Solak, Eren Imre, Dilek Gogas Yavuz

**Affiliations:** Department of Endocrinology and Metabolism, Marmara University School of Medicine, Istanbul 34899, Turkey; Department of Endocrinology and Metabolism, Marmara University School of Medicine, Istanbul 34899, Turkey; Department of Endocrinology and Metabolism, Marmara University School of Medicine, Istanbul 34899, Turkey

**Keywords:** Paget disease of bone, hypocalcemia, skull involvement, vitamin D deficiency, cranial enlargement, ^18^F-sodium fluoride PET/CT

## Abstract

Paget disease of bone (PDB) is a focal disorder of accelerated bone remodeling that typically presents with bone pain, deformity, or elevated alkaline phosphatase (ALP). Clinically significant hypocalcemia is uncommon and is usually associated with vitamin D deficiency and high bone turnover states. We report a 64-year-old woman who presented with altered mental status and severe hypocalcemia (total calcium 4.1 mg/dL [International System of Units (SI): 1.0 mmol/L]; reference range 8.5-10.1 mg/dL [SI: 2.1-2.5 mmol/L]) and markedly elevated ALP (1550 U/L; reference range 35-104 U/L). She had a history of progressive cranial enlargement and bilateral hearing loss. Laboratory evaluation revealed profound vitamin D deficiency. Skull radiography demonstrated extensive calvarial thickening with a classic “cotton wool” appearance, and bone scintigraphy showed intense tracer uptake throughout the cranial bones. ^18^F-sodium fluoride positron emission tomography/computed tomography confirmed diffuse osteoblastic activity confined to the calvarium without evidence of malignant skeletal lesions. Hypocalcemia resolved with calcium and vitamin D repletion, and intravenous zoledronic acid was administered for persistently active Paget disease. This case highlights that severe vitamin D deficiency is the primary driver of hypocalcemia, while high bone turnover may amplify its severity, underscoring the need for metabolic correction before antiresorptive therapy.

## Introduction

Paget disease of bone (PDB) is a chronic focal disorder of bone remodeling characterized by excessive osteoclastic resorption followed by disorganized osteoblastic bone formation [1, 2]. It predominantly affects older adults and may be monostotic or polyostotic, most commonly involving the pelvis, femur, spine, skull, and tibia [1-3]. Serum alkaline phosphatase (ALP) is often elevated and correlates with disease activity, although many patients remain asymptomatic and are diagnosed incidentally [2-4]. Pathogenesis involves abnormal, hypernucleated osteoclasts with increased resorptive capacity, influenced by genetic predisposition and environmental factors [5-7]. Symptomatic disease typically presents with bone pain, deformity, fractures, and site-specific complications related to the involved skeletal region [2-4].

Cranial involvement is a well-recognized manifestation and may result in progressive skull enlargement, headaches, cranial nerve compression, and hearing loss due to involvement of the temporal bone or auditory canal [1, 8, 9]. Cranial involvement in PDB has been reported in approximately 25-65% of cases across the literature [5, 8, 10]. Radiographically, skull disease classically demonstrates mixed lytic and sclerotic lesions producing the characteristic “cotton wool” appearance [1, 2]. Functional imaging with bone scintigraphy and positron emission tomography (PET) techniques may further delineate the metabolic activity and extent of disease [11-13].

Despite being one of the most common metabolic bone disorders in older populations, clinically significant metabolic disturbances at presentation are rare [1-3]. Hypocalcemia is particularly uncommon and usually occurs in the setting of extensive disease, high bone turnover, vitamin D deficiency, or following initiation of antiresorptive therapy [14-16].

Here we present an unusual case in which extensive cranial PDB, compounded by severe vitamin D deficiency, resulted in profound, life-threatening hypocalcemia requiring intensive metabolic support.

## Case presentation

A 64-year-old woman was brought to the emergency department for 1 week of progressive somnolence, psychomotor slowing, and dysarthria, accompanied by markedly reduced oral intake. Her medical history was notable for a hemorrhagic stroke 5 years earlier with residual functional dependence. She was not taking regular medications. Parental consanguinity (first cousins) was reported, without a known family history of metabolic or genetic disease. She had no prior fractures.

On arrival, vital signs were stable (blood pressure 130/70 mmHg, heart rate 75/min, temperature 36.7 °C, glucose 90 mg/dL [International System of Units (SI): 5.0 mmol/L]) (reference range 70-100 mg/dL [SI: 3.9-5.6 mmol/L]). She was disoriented with a Glasgow Coma Scale score of 11. Examination revealed prominent frontal and temporal bossing with increased head circumference (Fig. 1), reportedly progressive over 4 years. The family also described progressive bilateral hearing loss and recurrent headaches over the preceding 2 years. Chvostek sign was present. Electrocardiography demonstrated sinus rhythm with a prolonged corrected QT interval (QTc) of 490 ms (reference range <470 ms in women).

**Figure 1:**
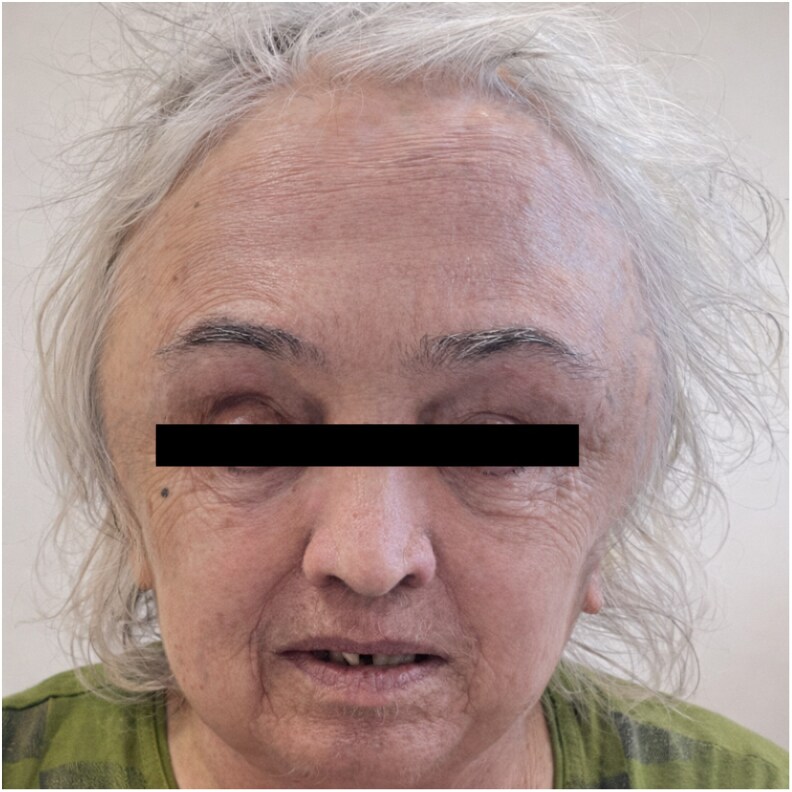
Progressive cranial enlargement in Paget disease of bone. Clinical photograph demonstrating marked frontal and temporal bossing with increased head circumference, reflecting long-standing cranial involvement in Paget disease of bone. The cranial enlargement reportedly progressed over approximately 4 years and was associated with bilateral hearing loss and recurrent headaches.

## Diagnostic assessment

Initial laboratory testing demonstrated profound hypocalcemia with total calcium 4.1 mg/dL (SI: 1.0 mmol/L) (reference range 8.5-10.1 mg/dL [SI: 2.1-2.5 mmol/L]) and ionized calcium 2.3 mg/dL (SI: 0.57 mmol/L) (reference range 4.6-5.3 mg/dL [SI: 1.15-1.33 mmol/L]), with phosphate 2.7 mg/dL (SI: 0.87 mmol/L) (reference range 3.0-4.5 mg/dL [SI: 0.97-1.45 mmol/L]), albumin 3.1 g/dL (SI: 31 g/L) (reference range 3.5-5.0 g/dL [SI: 35-50 g/L]), magnesium 1.6 mg/dL (SI: 0.66 mmol/L) (reference range 1.7-2.2 mg/dL [SI: 0.70-0.91 mmol/L]), and creatinine 0.6 mg/dL (SI: 53 µmol/L) (reference range 0.6-1.2 mg/dL [SI: 53-106 µmol/L]). ALP was markedly elevated at 1550 U/L (reference range 35-104 U/L). Arterial blood gas analysis showed a pH of 7.39 (reference range 7.35-7.45) and a bicarbonate level of 30 mmol/L (reference range 22-28 mmol/L).

Neuroimaging (brain computed tomography and diffusion-weighted magnetic resonance imaging) excluded acute ischemia or hemorrhage, and her neurologic findings were attributed to metabolic encephalopathy due to severe hypocalcemia. Endocrine evaluation revealed secondary hyperparathyroidism with parathyroid hormone (PTH) 225 pg/mL (SI: 23.8 pmol/L) (reference range 15-65 pg/mL [SI: 1.6-6.9 pmol/L]) and severe vitamin D deficiency with 25-hydroxyvitamin D <5 ng/mL (SI: <12.5 nmol/L) (reference range 30-100 ng/mL [SI: 75-250 nmol/L]). The patient's predominantly indoor lifestyle, limited sunlight exposure, and low dietary calcium intake likely contributed to the severity of vitamin D deficiency. A 24-hour urine collection showed hypocalciuria (urinary calcium 32 mg/day [SI: 0.8 mmol/day]; reference range 100-300 mg/day [SI: 2.5-7.5 mmol/day]). The calculated calcium/creatinine clearance ratio was 0.016, which was not consistent with familial hypocalciuric hypercalcemia and supported secondary hyperparathyroidism in the context of vitamin D deficiency.

Skull radiography demonstrated mixed lytic and sclerotic changes with a classic “cotton wool” appearance (Fig. 2). No radiographic features suggestive of osteomalacia (eg, looser zones or generalized demineralization) were identified on the available imaging studies. Technetium-99m bone scintigraphy showed diffuse, intense tracer uptake throughout the cranial bones, consistent with highly active disease; maximal skull thickness was approximately 6 cm. Fluorodeoxyglucose PET/computed tomography (CT) showed no evidence of primary malignancy or metastatic disease. For further characterization, ^18^F-sodium fluoride PET/CT (^18^F-NaF PET/CT) demonstrated diffuse, intense calvarial uptake (maximum standardized uptake value [SUVmax] 89.8) concordant with scintigraphy, consistent with markedly increased osteoblastic activity in PDB (Fig. 3), without NaF-avid lesions suggestive of malignant skeletal involvement. Given the characteristic imaging pattern and technical limitations for lesion localization, bone biopsy was deferred. Clinical exome sequencing performed using next-generation sequencing technology did not identify any pathogenic or likely pathogenic germline variants associated with PDB. The analysis included a targeted osteogenesis imperfecta and metabolic bone disease gene panel covering *BMP1, COL1A1, COL1A2, CRTAP, FKBP10, IFITM5, MBTPS2, MESD, P3H1, PPIB, SERPINF1, SERPINH1, SP7, SQSTM1, TNFRSF11A, TNFRSF11B, VCP,* and *WNT1*, with full exon and exon–intron boundary sequencing. No clinically significant variants were detected.

**Figure 2:**
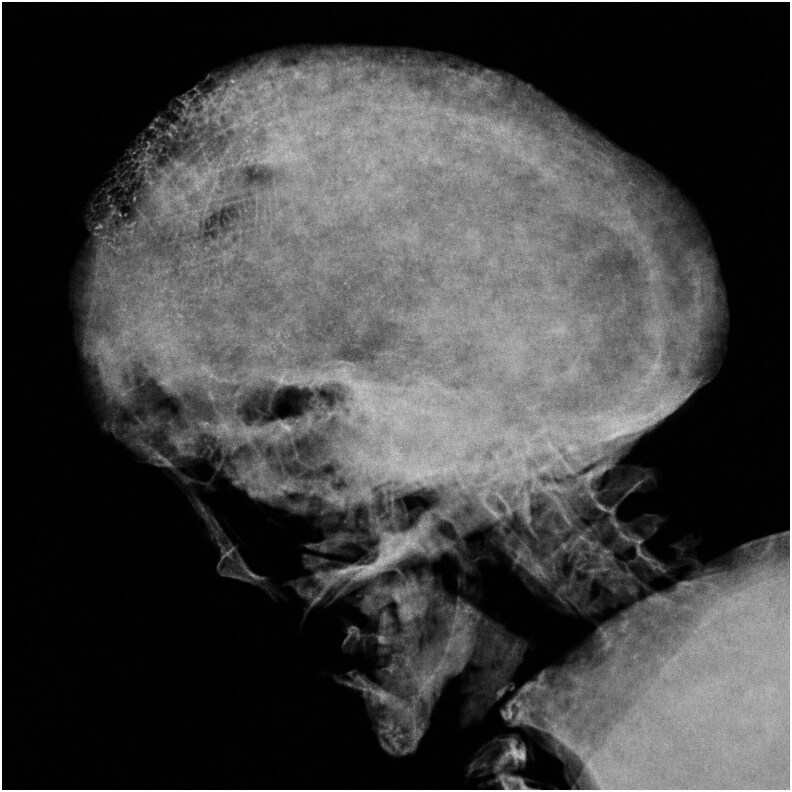
Skull radiography showing classic “cotton wool” appearance. Lateral skull radiograph demonstrating mixed lytic and sclerotic changes with patchy areas of sclerosis characteristic of advanced Paget disease of bone. The heterogeneous calvarial thickening produces the typical “cotton wool” appearance associated with active cranial involvement.

**Figure 3:**
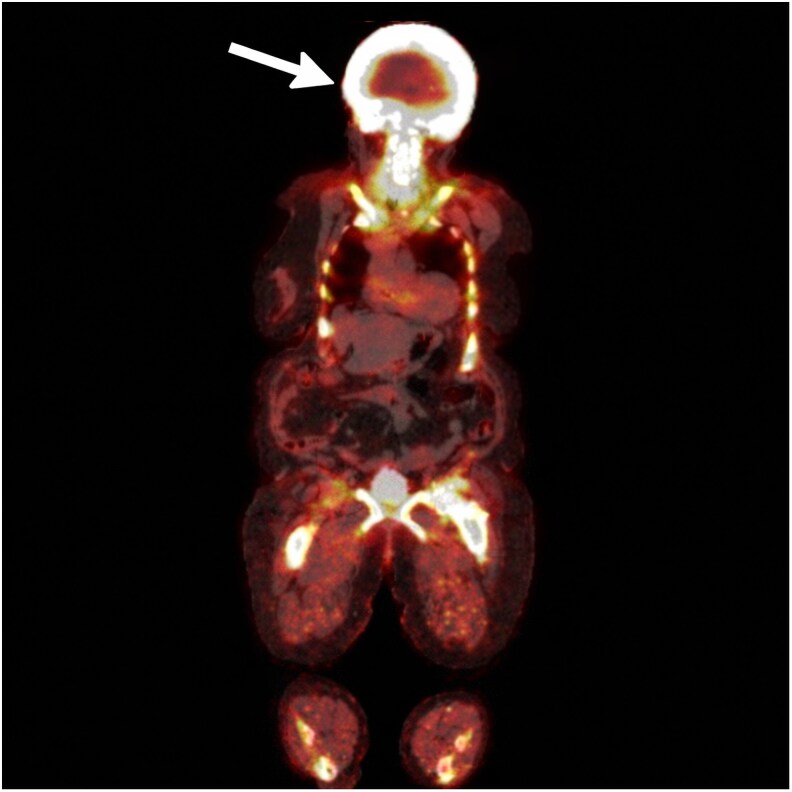
^18^F-sodium fluoride positron emission tomography/computed tomography (^18^F-NaF PET/CT) demonstrating diffuse, intense tracer uptake confined to the calvarium, indicative of markedly increased osteoblastic activity. The maximum standardized uptake value (SUVmax) reached 89.8, consistent with severe metabolic activity. No focal NaF-avid lesions suggestive of malignant skeletal involvement were identified, supporting the diagnosis of active Paget disease of bone rather than metastatic or infiltrative pathology.

## Treatment

The patient was treated with intravenous calcium gluconate and magnesium sulfate for acute electrolyte correction. Clinical and biochemical improvement was observed within 3-4 days of initiating therapy, allowing discontinuation of intravenous supplementation and transition to oral calcium carbonate and magnesium oxide.

Vitamin D replacement was initiated with 150 000 IU of intramuscular cholecalciferol, followed by 50 000 IU weekly until a cumulative loading dose of 300 000 IU was achieved. Oral calcitriol (0.5 µg/day) was temporarily administered during the acute phase and discontinued once serum 1,25-dihydroxyvitamin D increased to 90 pg/mL (SI: 216 pmol/L) (reference range 20-60 pg/mL [SI: 48-144 pmol/L]) to mitigate the risk of vitamin D–related toxicity. After completion of the repletion phase, the patient was transitioned to a maintenance regimen of 2000 IU/day of vitamin D (Table 1). The patient was discharged with instructions to continue oral calcium and vitamin D supplementation. She was counseled on improving dietary calcium intake and safe sunlight exposure, and follow-up visits were planned every 3 months with biochemical monitoring at least every 6 months.

**Table 1:** Serial biochemical parameters at admission and after correction of severe hypocalcemia, illustrating the response to calcium, vitamin D, and antiresorptive therapy

Parameter	At Admission	After treatment (3-month follow-up)	Reference range	Treatment administered
Calcium	4.1 mg/dL (1.0 mmol/L)	9.7 mg/dL (2.42 mmol/L)	8.5-10.1 mg/dL (2.1-2.5 mmol/L)	IV calcium gluconate; oral calcium carbonate
Phosphate	2.1 mg/dL (0.68 mmol/L)	3.8 mg/dL (1.23 mmol/L)	3.0-4.5 mg/dL (0.97-1.45 mmol/L)	Calcium and vitamin D repletion
PTH	125 pg/mL (13.3 pmol/L)	40.7 pg/mL (4.3 pmol/L)	15-65 pg/mL (1.6-6.9 pmol/L)	Calcitriol 0.5 µg/day
25-Hydroxyvitamin D	<5 ng/mL (<12.5 nmol/L)	32 ng/mL (80 nmol/L)	30-100 ng/mL (75-250 nmol/L)	IM → oral cholecalciferol
ALP	1550 U/L	1000 U/L	30-120 U/L	Zoledronic acid 5 mg IV
Magnesium	1.5 mg/dL (0.62 mmol/L)	2.0 mg/dL (0.82 mmol/L)	1.7-2.2 mg/dL (0.70-0.90 mmol/L)	IV → oral magnesium

Hypocalcemia and vitamin D deficiency were corrected within 3 months, whereas ALP remained elevated, reflecting persistent high bone turnover prior to zoledronic acid administration.

Abbreviations: 25(OH)D, 25-hydroxyvitamin D; ALP, alkaline phosphatase; IM, intramuscular; IV, intravenous; PTH, parathyroid hormone.

## Outcome and follow-up

The patient's mental status gradually returned to baseline as serum calcium levels normalized. Clinical improvement was observed within 3-4 days of initiating intravenous calcium and vitamin D replacement. The previously prolonged QTc improved on follow-up electrocardiography, and Chvostek sign resolved, confirming both biochemical and clinical recovery.

At the 3-month follow-up, calcium–phosphate metabolism had normalized, with PTH 40.7 pg/mL (SI: 4.3 pmol/L), total calcium 9.7 mg/dL (SI: 2.42 mmol/L), phosphate 3.8 mg/dL (SI: 1.23 mmol/L), albumin 4.0 g/dL (SI: 40 g/L), and magnesium 2.0 mg/dL (SI: 0.82 mmol/L). Serum 25-hydroxyvitamin D increased to 33 ng/mL (SI: 82.5 nmol/L), consistent with adequate repletion. However, ALP remained elevated at 1000 U/L, consistent with persistent high bone turnover. Normalization of serum calcium at 3 months was attributed to vitamin D repletion and improved calcium intake; zoledronic acid was administered subsequently to treat persistently active PDB as reflected by elevated ALP. Given ongoing biochemical evidence of active Paget disease, the patient received a single 5-mg intravenous infusion of zoledronic acid. Zoledronic acid was administered only after confirmation of stable serum calcium levels, in order to reduce the risk of bisphosphonate-associated hypocalcemia. The infusion was well tolerated, with no adverse events. Serum calcium levels remained stable after zoledronic acid infusion, with no recurrence of hypocalcemia, likely reflecting prior correction of vitamin D deficiency and continued calcium and vitamin D supplementation.

## Discussion

This case highlights an uncommon metabolic presentation of PDB, in which massive cranial involvement combined with severe vitamin D deficiency resulted in life-threatening hypocalcemia. Although PDB is relatively frequent in older adults, most patients are asymptomatic or present with localized skeletal manifestations (bone pain, deformity, or fracture risk), rather than severe systemic metabolic complications [1-4].

The mechanism of hypocalcemia in this patient was likely multifactorial. Severe vitamin D deficiency was the primary driver, leading to impaired intestinal calcium absorption and secondary hyperparathyroidism. Paget disease is characterized by markedly increased osteoclastic resorption followed by excessive, disorganized osteoblastic bone formation, resulting in accelerated bone turnover [5, 6, 15, 17]. In extensive, highly active disease, calcium may be rapidly incorporated into newly formed bone, creating a functional “calcium sink” [14, 15]. In this context, the markedly increased bone turnover may have further contributed to the severity of hypocalcemia through a mechanism resembling a hungry bone–like state, particularly in the setting of profound vitamin D deficiency and limited dietary calcium intake. Thus, while vitamin D deficiency was the principal cause of hypocalcemia, active Paget disease may have exacerbated the depth and clinical severity of the calcium imbalance.

Imaging was central for confirming diagnosis, defining extent, and excluding malignancy. Skull radiography demonstrated the classic “cotton wool” appearance of advanced cranial PDB [1, 2], and technetium-99m bone scintigraphy showed diffuse intense uptake throughout the cranial bones, consistent with markedly increased osteoblastic activity. ^18^F-NaF PET/CT further demonstrated diffuse tracer uptake confined to the calvarium without malignant skeletal lesions, supporting active pagetic bone remodeling. ^18^F-NaF PET/CT has high sensitivity for osteoblastic activity and may better delineate disease extent compared with conventional scintigraphy in selected cases [11-13]. In this patient, the concordant benign uptake pattern and absence of aggressive features supported deferral of bone biopsy, particularly given the technical challenges of skull lesion targeting.

Cranial PDB may mimic infiltrative or malignant disease when accompanied by neurologic symptoms and dramatic radiographic changes [8, 9, 12]. While skull enlargement, headaches, and hearing loss are well-recognized complications, profound derangements in calcium homeostasis are distinctly rare [1, 8, 14]. The approximately 6-cm skull thickness observed in this case reflects an exceptionally high disease burden.

Management followed a staged approach consistent with guideline-based care [2-4]. Initial treatment prioritized aggressive correction of metabolic abnormalities with calcium and vitamin D repletion. This step is critical because initiating potent antiresorptive therapy in the presence of vitamin D deficiency may precipitate or worsen hypocalcemia [15, 16, 18]. After stabilization of calcium metabolism, intravenous zoledronic acid was administered, which is first-line therapy for active PDB and is associated with durable biochemical remission in most patients [2, 3, 10, 18, 19].

Despite biochemical improvement, cranial enlargement and hearing loss persisted, consistent with evidence that established deformity and compressive sequelae may be irreversible even after suppression of disease activity [2, 8, 9].

In summary, this case expands the clinical spectrum of PDB and reinforces key principles: (i) severe vitamin D deficiency (with or without low calcium intake) should be considered a primary cause of hypocalcemia in older adults; (ii) in patients with progressive cranial deformity and characteristic skull imaging with markedly elevated ALP, concomitant PDB should be evaluated; (iii) safe management requires correction of metabolic abnormalities before antiresorptive therapy; and (iv) ^18^F-NaF PET/CT can provide valuable complementary information on disease activity and distribution in complex presentations [11-13]. Differential diagnoses for marked cranial sclerosis, including fibrous dysplasia and osteopetrosis, should also be considered based on clinical and imaging context.

## Learning points

- Severe vitamin D deficiency—often compounded by low dietary calcium intake—is a common and important cause of hypocalcemia in older adults.
- High bone turnover states, such as extensive Paget disease, may exacerbate the severity of hypocalcemia in the presence of profound vitamin D deficiency by functioning as a skeletal “calcium sink.”
- In patients with progressive cranial enlargement, characteristic “cotton wool” skull changes, and markedly elevated ALP, Paget disease should be evaluated.
- Management should follow a staged approach: correction of calcium and vitamin D abnormalities must precede potent antiresorptive therapy to prevent treatment-related worsening of hypocalcemia.
- Long-term care requires maintenance vitamin D supplementation, adequate dietary calcium intake, safe sunlight exposure, and regular biochemical monitoring to prevent recurrence and ensure metabolic stability.

## Contributors

D.G.Y., Y.G.S., and E.I. were involved in the diagnosis and clinical management of this patient. D.G.Y. conceptualized the case report. Y.G.S. performed data collection and literature review. D.G.Y. and Y.G.S. drafted the original manuscript. E.I. contributed to patient follow-up and critical revision of the manuscript. All authors reviewed and approved the final version for publication.

## Data Availability

All data generated or analyzed during this study are included in this published article.
